# The effectiveness of an online intervention in stimulating injury-preventive behaviour in adult novice runners: Results of a randomised controlled trial

**DOI:** 10.17159/2078-516X/2021/v33i1a11297

**Published:** 2021-09-20

**Authors:** E Kemler, MH Cornelissen, V Gouttebarge

**Affiliations:** 1 Dutch Consumer Safety Institute, Amsterdam, The Netherlands; 2 Amsterdam UMC, University of Amsterdam, Department of Orthopaedic Surgery, Amsterdam Movement Sciences, Meibergdreef 9, Amsterdam, The Netherlands; 3 Section Sports Medicine, Faculty of Health Sciences, University of Pretoria, Pretoria, South Africa; 4 Amsterdam Collaboration on Health & Safety in Sports (ACHSS), Amsterdam UMC IOC Research Center of Excellence, Amsterdam, The Netherlands

**Keywords:** behaviour, running, primary prevention, tailor-made, intervention

## Abstract

**Background:**

The online intervention Runfitcheck was developed to stimulate injury-preventive behaviour among adult novice runners.

**Objectives:**

This study evaluated the effectiveness of Runfitcheck on injury-preventive behaviour among adult novice runners.

**Methods:**

A randomised controlled trial was conducted among adult novice runners. The intervention group had access to the Runfitcheck intervention, the control group performed their running activities as usual. One, three, and five months after enrolment, participants reported retrospectively what they had done regarding injury-preventive behaviour (operationalised as (i) using a (personalised) training schedule; (ii) performing strength and technique exercises; and (iii) performing a warm-up routine prior to running). Relative Risks (RR) and 95% Confidence Interval (95%CI) were used to analyse behavioural change.

**Results:**

The intervention group (n=715) searched more often for information about a warm-up routine (RR 1.211; 95%CI 1.080–1.357), and added more often strength exercises to their warm-up routine (RR 1.228; 95%CI 1.092–1.380). The intervention group performed more often running technique exercises compared to the control group (n=696) (RR 1.134; 95%CI 1.015–1.267), but less often strength exercises (RR 0.865 (95%CI 0.752–0.995). Within the group of runners that did not perform any warm-up routine at enrolment (n=272), the intervention group started to perform a regular warm-up routine more often than the control group (RR 1.461; 95%CI 1.084–1.968). No significant results were found for using a training schedule.

**Conclusion:**

The online intervention Runfitcheck was effective in stimulating aspects of injury-preventive behaviour in adult novice runners, mostly related to a warm-up routine.

**Trial registration:**

NL6225, Registered April 24^th^ 2007 - Retrospectively registered, https://www.trialregister.nl/trial/6225

Running is one of the five most popular sports activities among adults worldwide and one of the most favourite sports for starting to become physically active^[1]^. In the Netherlands, around 30% of the running population consists of novice runners who have less than one year of running experience^[2]^.

In addition to its beneficial health effects, running is also associated with a high risk of musculoskeletal injuries. The incidence of running-related injuries (RRIs) is reported to range from three to 59 injuries per 1 000 exposure hours.^[3–5]^ In particular, novice runners are at high risk of sustaining a RRI, especially of the lower extremities.^[2,3,6]^ Risk factors for RRIs have been extensively investigated, but evidence remains contradictory and inconclusive. A history of previous injury in the past 12 months is reported to be the main risk factor for RRIs.^[7,8]^ According to several review articles, half of all RRIs in runners are related to training errors.^[9,10]^ Furthermore, goal-setting seems to be of more importance to runners than a realistic training load. Sports goal-oriented running and especially running in order to complete a certain distance plus participating in an event is associated with a higher risk of a RRI.^[11]^

RRIs among novice runners could be averted by favourable injury-preventive behaviour, such as modifying the training load.^[9,10]^ However, novice runners might not be able to assess their training load properly, are probably not aware of the training errors they make, or simply ignore the signals their body gives due to their goal-setting behaviour in running.^[11]^ To stimulate favourable injury-preventive behaviour in novice runners, such as performing a warm-up and cool-down routine, adjusting running volume/intensity, and responding to body signals (listening to your body), some interventions have recently been developed and have been used by runners with promising results, leading even to the prevention of RRIs.^[12,13]^ Hespanhol et al. showed that online tailored injury-preventive advice led to a reduction of RRIs among trail runners.^[12]^ The intervention developed by Adriaensens et al. was effective in stimulating injury-preventive behaviour among runners^[13]^, but was time-consuming.

A new online intervention (‘Runfitcheck’) was developed to stimulate injury-preventive behaviour among novice runners. The objective of this study was to evaluate the effectiveness of Runfitcheck on injury-preventive behaviour among adult novice runners.

## Methods

### Study design and participants

A randomised-controlled trial (RCT), with a follow-up period of five months (March 2017 – July 2017), was conducted, consisting of an intervention and control group. The design of this study is described in detail elsewhere.^[14]^ The study design and protocol were approved by the Medical Ethics Review Committee of the Academic Medical Center (W16_335 # 16.417, Amsterdam, The Netherlands). The trial is registered in the Dutch Trial Registry (ID: NL6225).

The group of participants consisted of adult novice runners. Inclusion criteria were: (i) aged 18 years and older; (ii) having less than one year of running experience and/or not considering themselves as an experienced or very experienced runner. The choice for the combination of a time definition (less than one year of experience) and a definition based on feelings (not considering themselves as an experienced or very experienced runner) was made in accordance with running experts to concur with the Dutch context. Participants were recruited via social media networks (Facebook, various websites, Twitter, LinkedIn, newsletters) of the participating organisations. Participants who completed all questionnaires were entered into a draw offering the possibility of winning a gift voucher to the value of €200 (±R3 500) for running clothes. All participants of the study provided informed consent online.

### Protocol

Participants within the intervention group obtained access to the Runfitcheck intervention.^[13]^ No further conditions were applied to the use of the intervention. The Runfitcheck intervention was developed according to an evidence-based (Knowledge Transfer Scheme and Intervention Mapping) and practice-based (running experts) approach to stimulate injury-preventive behaviour among novice runners. More information on the development process and content of the Runfitcheck is described in detail elsewhere^[15]^ and in Afc A. The participants in the control group performed their running activities as usual.

The main outcome measure of the study was injury-preventive behaviour by means of: (i) using a (personalised) training schedule; (ii) performing strength and technique exercises^[16]^; and (iii); performing a warm-up prior to running.^[17]^ Each of these injury-preventive behaviours was divided into preparatory and executional actions:

- the training schedule consisted of two preparatory and one executional action, namely, searching for a training schedule, creating a personal training schedule, and using a general training schedule;
- strength and technique exercises consisted of two preparatory and two executional actions, namely, searching for both strength and technique exercises and executing both types of these exercises;
- the warm-up consisted of one preparatory and two executional actions: searching for information about a warm-up routine for runners, performing a warm-up routine (extensive or otherwise), and adding strength exercises to a warm-up routine. An extensive warm-up is a warm-up routine in which the runner starts at a slow pace, performs strength exercises and sport-specific exercises. All injury-preventive behaviours were assessed through single answer questions from a pre-determined set of responses (Yes/No/Not applicable).

Participants were asked to fill in four online questionnaires (T0-T3). At enrolment (T0), participants were asked to report the injury-preventive behaviour (warm-up routine, strength and technique exercises, use of a (personalised) training schedule)) they usually performed before or during their running activities. Additionally, participants were asked about their demographic characteristics (age in years, gender), running experience (in months), frequency per week of running and other sports activities in the previous three months, as well as any other injury-preventive behaviour. One month after enrolment (T1), three months later (T2), and five months later (T3), participants were asked to retrospectively report in detail, via an online questionnaire, what they had done in that time frame (one month between T1 and T0, two months between T2 and T1, two months between T3 and T2, respectively) in terms of preparatory and executional actions during their running activities.

In previous literature, a 13% increase in injury-preventive behaviour among recreational adult runners (in this case, the inclusion of a warm-up) was found during a three-month follow-up period.^[13]^ Therefore, in this study, it was hypothesised that the Runfitcheck intervention could lead to a 10% difference in favourable injury-preventive behaviour in the intervention group in comparison to the control group. A choice was made to use the word ‘difference’ instead of ‘increase’, as a difference between the two groups was considered as more important than only an increase. For example, if more runners in the intervention group execute favourable injury-preventive behaviour, but runners in the control group change their behaviour too, an increase will be found, but this is unlikely to be a statistically significant difference.

To achieve 80% power with a significance level of 0.05, the sample size calculation revealed that 384 participants per study group were needed in this study. Considering a response rate of 85% and a drop-out rate of 10% over the five months follow-up period, a total of at least 1 000 participants (500 per study group) in this study needed to be approached.

As our main outcome measure for injury-preventive behaviour the study was divided into three different behaviours with several preparatory and executional actions, participants could perform one part of the outcome measure (e.g. performing a warm-up routine), while they did not perform the other behaviours. Hence, after T0, all eligible participants were included in the study and allocated at random to either the intervention or control group, using a computerised random number generator (the Aselect function in Excel). No restrictions were imposed to achieve a balance between the groups in size or characteristics for the allocation, and simple randomisation was performed. Also, concealed allocation was used. All steps in the randomisation process were performed by the principal researcher. Neither the participants in the intervention group nor the researchers were blinded in this study.

### Statistical analysis

Descriptive analyses (mean, standard deviation, frequency) were conducted for the different baseline variables in both study groups. Baseline variables were analysed for differences between the intervention and control groups (chi-square test, independent T-tests).

For the executional actions, structural behavioural change was evaluated. A behaviour change is regarded as structural if runners changed their behaviour at a certain point in time, and continued to execute the behaviour until the end of the follow-up period, or if runners executed the behaviour at baseline, and continued to execute it until the end of the follow-up period.

The relative risk (RR) and 95% confidence interval (95% CI) were calculated using the risk estimates within the chi-square analyses (only available for a 2×2 table) and were used to analyse behavioural change in the preparatory and executional injury-preventive actions between T0 and T3. Analyses were performed according to the intention to treat analyses: (i) using a (personalised) training schedule; (ii) performing strength and technique exercises; (iii) performing a warm-up routine (extensive or otherwise). Participants were included in the study until they dropped out, or after completing all four questionnaires. Missing data were not included.

For the analyses, those participants who executed the desired behaviour at baseline, and those participants who did not execute this behaviour at enrolment but did execute it during the follow-up period were grouped together and compared with participants who did not execute the desired behaviour at enrolment and who did not start or execute this behaviour during the follow-up period.

In the sub-analyses, participants were only included if they did not perform the favourable injury-preventive behaviour at enrolment. Relative risks and 95% CI were performed to reveal the ‘actual effect’ of the intervention on injury-preventive behaviour.

For all analyses, significance was accepted at p<0.05.

## Results

**Fig. 1:**
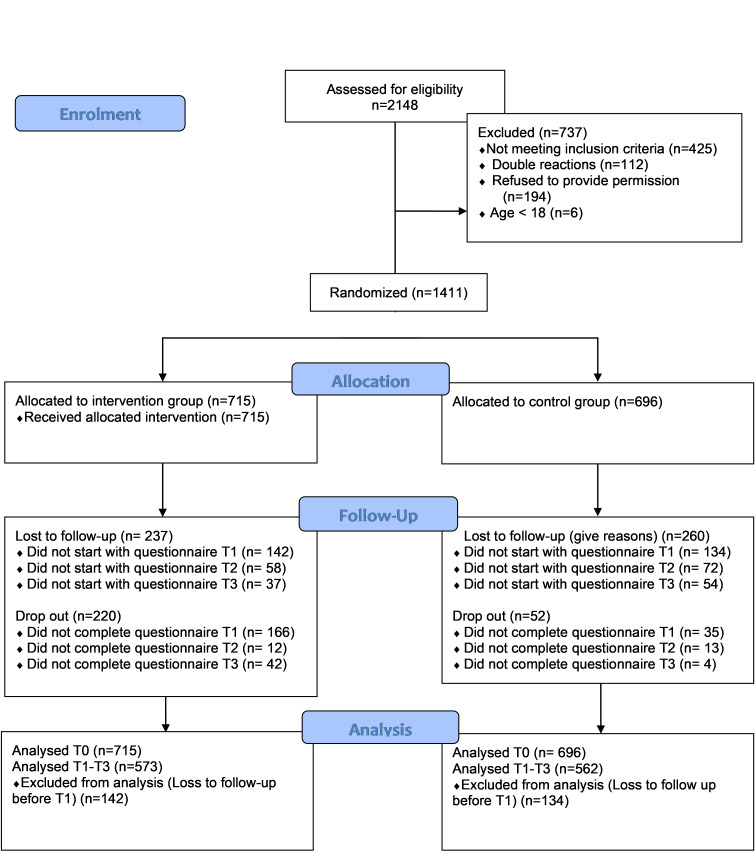
Flow chart of the participants of the randomised prospective controlled trial.

In total, 2 148 participants were interested in participating in the study, of whom only 1 411 were eligible for participation according to the inclusion criteria. Of these eligible participants, 715 were randomly allocated to the intervention group and 696 to the control group. Eighty percent of the participants (n=1 135) completed at least one of the follow-up questionnaires and were therefore included in the analyses. Almost half of the participants completed all questions in all four questionnaires (46%; n=642). A complete flowchart of the participants is shown in Fig. 1.

Of the 1 411 participants, 73% (n=1 025) were female, and the mean age was 38.1 years (SD=10.4; Table 1). Almost one-third of the participants had less than one year of running experience (30%). In the three months prior to the study, 14% of the participants had run less than once per week while 21% had run once per week on average. Sixty-six percent had run at least twice per week.

At baseline, 81% of the intervention group reported that they performed some kind of warm-up routine at the start of their training session, 19% performed an extensive warm-up routine in which they started to run at a slow pace and performed strength and sport-specific exercises. In the control group, 80% performed some kind of warm-up routine, while 20% performed an extensive warm-up routine. A general training schedule was used by 43% of the intervention group and 43% in the control group, and a personalised training schedule by 18% in the intervention group and 17% in the control group. More than half of the intervention group performed strength exercises (56%) and 31% performed exercises to improve their running techniques. In the control group, 56% performed strength exercises and 28% performed exercises to improve their running techniques. There were no significant differences between the two groups.

After five months of follow-up, it turned out that intervention group searched more often for information about a warm-up routine (56% versus 45%; RR 1.211 (95% CI 1.080–1.357); Table 2), and added more often strength exercises to their warm-up routine (49% versus 38%; RR 1.228 (95% CI 1.092–1.380)). The intervention group performed running technique exercises more often compared to the control group (59% versus 52%; RR 1.134 (95% CI 1.015–1.267)), but less often strength exercises (72% versus 78%; RR 0.865 (95% CI 0.752–0.995)).

### Sub-analyses

After five months of follow-up, within the group of runners that did not perform any warm-up routine at the start of the study (n=272; 70% female, mean age 35.8 years (SD 9.3)), the intervention group searched more often for information on a warm-up routine (54% versus 34%; n=194; RR 1.444 (95% CI 1.098–1.901)), performed a regular warm-up routine more often than the control group (47% versus 28%; n=196; RR 1.461 (95% CI 1.084–1.968)), and added strength exercises to their warm-up routine more often than the control group (33% versus 17%; n=195; RR 1.504 (95% CI 1.039–2.179) Table 3).

Analyses within the group of runners that did not perform an extensive warm-up routine at the start of the study (n=882; 71% female, mean age 38.1 years (SD 10.2)) revealed similar results. The intervention group searched more often for information concerning a warm-up routine (56% versus 45%; n=859; RR 1.222 (1.083–1.380)), performed a regular warm-up routine more often than the control group (53% versus 40%; n=882; RR 1.257 (95% CI 1.112–1.421)), and added strength exercises to their warm-up routine more often (43% versus 30%; n=880; RR 1.290 (95% CI 1.127–1.478)) than similar runners in the control group.

Analyses within the group of runners that did not perform any running technique exercises at the start of the study (n=691; 72% female, mean age 38.7 years (SD=10.3)) revealed that the intervention group performed these exercises more often than the control group (41% versus 31%; RR 1.208 (95% CI 1.042–1.400)).

Analyses within the group of runners that did not perform any strength exercises at the start of the study (n=426; 72% female, mean age 38.7 years (SD=10.3)) revealed that the intervention group performed these exercises less often than the control group (37% versus 50%; n=426; RR 0.790 (95% CI 0.669–0.932)).

Runners may have added strength exercises to their warm-up routine, or performed strength exercises at some other point in time during a week. The analysis showed that among those runners who did not perform any strength exercises at baseline (n=424), the intervention group added strength exercises to their warm-up routine more often (22% versus 11%), while the control group started to perform strength exercises at some other point of time during a week more often (30% versus 18%) (Pearson’s chi-square 13.55, p=0.004). In both the intervention and control groups, around 19% added strength exercises to their warm-up routine and started to perform strength exercises at some other point in time during a week. Forty percent in both groups did not perform any strength exercises at all.

With regard to the use of a (personalised) training schedule, there were no significant differences.

## Discussion

In this study, the effectiveness of the online intervention Runfitcheck in stimulating injury-preventive behaviour was evaluated among adult novice runners. Similar results were found in analyses in which the whole study population was included, and in analyses in which runners were included who did not perform a specific type of injury-preventive behaviour at enrolment. Performing a warm-up routine at the start of a training session was one of the important elements of the Runfitcheck intervention. In this intervention, the injury-preventive advice on a warm-up consisted of a short introduction on the benefits of performing a warm-up routine, followed by an instruction video with a voice-over of a warm-up routine for runners (lasting five minutes). The video was immediately accessible on the runner’s mobile phone, tablet or computer. Providing an easily accessible video thus seems to be effective in stimulating favourable injury-preventive behaviour. However, several aspects of the results with regard to the warm-up routine need to be addressed. A high percentage (81%) of runners performing any kind of warm-up routine at baseline might have made it difficult to identify the effect of the intervention as only 20% could benefit from it. This might have led to increasing ceiling effects of the intervention. However, with a total of 2.1 million runners in the Netherlands in 2013, of whom 620 000 were novice runners^[2]^, we believe that many runners could benefit from the intervention.

As previously mentioned, 81% of the runners performed some kind of warm-up routine at baseline. The details of regular and extensive warm-ups were starting at a slow pace, stretching, and sport-specific exercises. As stretching is not regarded as beneficial for injury prevention in runners ^[10,17,18]^, it is arguable how many of these 81% actually performed this in their warm-up routine. For this reason, we performed other analyses with those runners who did not perform an extensive warm-up routine at baseline. These analyses showed that runners in the intervention group who did not perform an extensive warm-up at baseline performed a regular warm-up routine more often than runners in the control group (53% versus 40%; n=882; RR 1.257 (95% CI 1.112–1.421)). They also added strength exercises to their warm-up routine more often (43% versus 30%; n=880; RR 1.290 (95% CI 1.127–1.478) when compared to similar runners in the control group. Although we do not know the quality of the warm-up routine that the runners performed, we do know that around 80% of the runners in the intervention group added strength exercises to this routine, as did 75% of the runners in the control group. For future studies, we believe it is important to define what a warm-up routine should be or put more effort in determining the quality of a warm-up routine (e.g. by questioning in more detail what runners do or have changed in their warm-up routine).

In contrast to injury-preventive aspects of a warm-up routine, the intervention group performed strength exercises less often than the control group. The results with regard to the inclusion of strength exercises surprised the research team, especially as the intervention group added more strength exercises to their warm-up routine. A possible explanation for this result might be that in the online questionnaires for both the intervention and control groups, the questions related to this topic were not completely identical. The intervention group was asked whether they had started to perform the strength exercises available in the intervention. Information on the performance of other strength exercises was, unfortunately, not collected. The control group was also asked if they performed any kind of strength exercises. This could have influenced the results of this part of the study. Also, an additional analysis revealed that the moment the runners performed the strength exercises, caused the difference between the two groups. Runners in the intervention group who did not perform any strength exercises at baseline added strength exercises to their warm-up routine more often (22% versus 11%), while runners in the control group started to perform strength exercises at some other point more often in time (29% versus 18%) (Pearson’s chi-square 13.546, p=0.004). Forty percent in both groups did not perform any strength exercises. Although these additional analyses showed that the negative outcome of the intervention with regard to strength exercises is probably actually not that negative at all while a positive outcome could not be demonstrated either.

In addition to the positive effects of their warm-up routine and running technique exercises, runners in the intervention group were requested more often to search for more information on injury prevention in running. They reported visiting two Dutch websites, one for the Dutch Consumer Safety Institute which consisted of information on the prevention of sports injuries, including RRIs (23% versus 8%; RR 1.964 95% CI 1.523–2.534), and a website of the Royal Dutch Athletics Association (KNAU), compared to runners in the control group (29% versus 18%, RR 1.365 95% CI 1.162–1.605). These websites were accessible in the Runfitcheck intervention via direct links to the specific websites.

For the preparatory and executional actions in the training schedules, no differences were found between the intervention and control groups. If we did find any differences, they were possibly difficult to interpret as negative or positive. Training errors are mentioned as a main cause of RRIs^[9,10]^, although a recent review found that very limited evidence exists to support the notion that changes (increases and decreases) in training load are associated with injury development^[18]^. Fields et al. stated in their review that excessive mileage and changes in training schedule are associated with an increased incidence of RRIs. Since each person’s body responds differently to the stress caused by running, individualised training programmes are recommended^[10]^. Linton and Valentin, on the contrary, found in their study that in the first year of running, runners using a self-devised training programme were more likely to be injured compared with runners using a structured programme ^[19]^. Although a self-devised training programme is not the same as a personalised training programme, it is difficult to determine what a good programme or training schedule is for a runner. Furthermore, we do not have enough detailed information to judge the training schedules the runners in our study used. We do know that it was either a personalised schedule or a regular training schedule, but we do not know the exact content of the schedules used, which is a limitation in our study.

Several other methodological considerations of our study can be addressed. Firstly, we included novice runners in our study identified according to a combination of two definitions: one based on time (less than one year of experience), and one based on feelings (not considering themselves as an experienced or very experienced runner). When developing the research design, the definition of novice runners was extensively discussed with running experts of the Royal Dutch Athletics Federation. The research group, together with these running experts, believed the definition based on time was too narrow and did not fit well enough with the Dutch running population. Hence, we used a combined definition for a novice runner. However, as we do not know how and why runners judge themselves as they did, our results could have been influenced by the use of our definitions. For further studies, and the design of injury preventive programmes, it would be of interest to explore how and why sports participants, including runners, judge themselves as they do

Secondly, the original sample size calculation revealed that at least 1 000 novice runners were needed to be enrolled in the study. Achieving such a high number of participants was challenging. Several methods were used to enhance enrolment, such as social media (Facebook, LinkedIn and Twitter), newsletters from the KNAU (digital) and the magazine Runner’s World (in print and digital), and the possibility to pre-register for the study. Furthermore, participants who completed all questionnaires were entered into a draw in which they could win a gift voucher to the value of €200 (±R3 500) for running clothes. The methods worked, since 2 148 volunteered for the study, of whom 1 411 were eligible. However, the adherence to the study after five months of follow-up was relatively low (46%). The draw to win a voucher for running clothes could be a possible reason for the participants’ low adherence rate (only wanting to participate to win the voucher, but not to complete the study). Another reason for low adherence could be the running population itself, in particular novice runners. An important aspect of being a novice runner or novice athlete is the aspect of being “unconscious incompetent”, referring to the first stage of the ‘four stages of competence’ model. ^[20]^ Novice athletes, or at least most of them, do not understand or know how they can prevent injuries and do not necessarily recognise the importance of prevention. As this trial focused on changing injury-preventive behaviour in novice runners, they might have denied the usefulness of the intervention and study and dropped out.

Additional analysis, however, did not reveal relevantly significant differences in characteristics between runners who dropped out of the study or who were lost to follow-up, and those who completed all questionnaires. Therefore, we still consider our results as meaningful.

Finally, we want to address the potential impact of recall bias on the calculation of running exposure. To define the running exposure of participants, we gathered information on running frequency. Running frequency is, however, not the sole outcome measure for running exposure. To get more insight into running exposure, it is important to gather information on running duration and/or distance as well as frequency. The potential of recall bias prevented us from doing so in this study.

Also in our study, at T2 and T3, participants had a two-month recall period, causing a possible recall bias. A recall period of one–three months is recommended for injury questionnaires^[21]^, similar to that used in our study. However, we focused on injury-preventive behaviour which could be more problematic to recall. Hence, a shorter recall period or a prospective study is advised for future studies, but researchers should be aware of the balance between research load for participants (and possible drop-out) and (lack off) recall bias.

A strength of our study is the design used. A RCT, when well designed, provides the strongest evidence of any epidemiological study design, and is usually used to evaluate the effectiveness of an intervention in an experimental setting. In this study, however, we did not evaluate the effectiveness of the Runfitcheck intervention in an experimental setting, but in a real-world setting, which in our opinion is another strength of the study. It is well-known that it is difficult to transfer interventions whose efficacy has been proved into real-world settings (efficacy versus effectiveness)^[22]^. With the development of Runfitcheck, the research group made the assumption that an increase in injury-preventive behaviour will ultimately lead to a decrease in RRIs. Our main focus was therefore on stimulating injury-preventive behaviour rather than preventing RRIs, as adjustment of behaviour is crucial before prevention of RRIs is even possible. In our study, participants in the intervention group were given access to the Runfitcheck intervention, but no further conditions were applied to the use of the intervention. We demonstrated effects of the Runfitcheck intervention in stimulating some aspects of injury-preventive behaviour among adult novice runners, indicating that an intervention like Runfitcheck actually could work in ‘the real world’.

Although we did find some positive outcomes, it is still unclear whether the results of our intervention with benefits of a warm-up routine are clinically relevant, and if these are enough to prevent RRIs. As mentioned in the introduction, RRIs among novice runners could be prevented by favourable injury-preventive behaviour such as modifying the training load ^[9,10]^. In our intervention we tried to focus on the physical load-taking capacity of runners and the motivation of runners to achieve their running goals to stimulate runners to modify their training load when necessary. Performing a warm-up routine was one of our suggestions. Although we did stimulate injury-preventive behaviour, this might not be enough to prevent RRIs. The transition from injury-preventive behaviour to the prevention of RRIs needs to be addressed in another randomised controlled trial.

The starting point of the development of the Runfitcheck intervention was a potentially effective but time-consuming – and therefore unattractive and complex – intervention for injury prevention in running^[13]^, and information on the number and severity of running-related injuries in the Netherlands. Adriaensens et al. developed a tailor-based online injury-prevention intervention (website) with informational videos about the aetiology and mechanisms of RRIs, combined with injury-preventive advice, and an online questionnaire. This online questionnaire allowed the website to provide tailored feedback based upon a series of predefined questions that create a personal risk profile of the user^[13]^. A 13% increase in injury-preventive behaviour (in this case, the inclusion of a warm-up) was found over a three-month follow-up period.^[13^ Although the intervention developed by Adriaensens et al. was effective, the online questionnaire for tailored feedback was time-consuming. Therefore, the Dutch Consumer Safety Institute developed the Runfitcheck intervention to encourage injury-preventive behaviour among novice runners without the associated time burden and was indeed able to induce a 10% difference in several aspects of injury-preventive behaviour in runners in favour of the intervention group. Furthermore, the results of this study showed that the realisation of a difference of 10% in injury-preventive behaviour is feasible using an online intervention.

## Conclusion

The online intervention Runfitcheck was effective in stimulating aspects of injury-preventive behaviour in adult novice runners, mostly related to a warm-up routine. The realisation of a 10% difference in favourable injury-preventive behaviour is feasible with an online intervention.

## Supplementary Information



## Figures and Tables

**Table 1:** Baseline characteristics of the participants (n=1 411)

Characteristic	Intervention group (n=715)	Control group (n=696)	Total (n=1 411)
**Gender, male**	192 (26.9%)	194 (27.9%)	386 (27.4%)
**Mean age in years (SD)**	38.2 (10.5)	37.9 (10.3)	38.1 (10.4)
**Running experience**			
<6 months	71 (9.9%)	61 (8.8%)	132 (9.4%)
6–12 months	140 (19.6%)	147 (21.1%)	287 (20.3%)
12–18 months	121 (16.9%)	113 (16.2%)	234 (16.6%)
18–24 months	105 (14.7%)	98 (14.1%)	203 (14.4%)
> 24 months	278 (38.9%)	277 (39.8%)	555 (39.3%)
**Running frequency in previous three months**			
Less than once per week	105 (14.7%)	87 (12.5%)	192 (13.6%)
Once per week	137 (19.2%)	157 (22.6%)	294 (20.8%)
Twice per week or more	473 (66.2%)	452 (64.9%)	925 (65.6%)
**Sport frequency in previous three months other than running**			
Not active in other sports	134 (18.7%)	109 (15.7%)	243 (17.2%)
Less than once per week	140 (19.6%)	121 (17.4%)	261 (18.5%)
Once per week	183 (25.6%)	194 (27.9%)	377 (26.7%)
Twice per week or more	258 (36.1%)	272 (39.1%)	530 (37.6%)

Data expressed as n(%) unless indicated otherwise.

**Table 2:** Preparatory injury-preventive actions and executional injury-preventive actions undertaken by all runners over five months of follow-up

	Intervention group	Control group	RR (95% CI)
**Using a (personalised) training schedule**			
Searched for a training schedule (n=970)	339 (79.8%)	420 (77.1%)	1.071 (0.941–1.218)
Created a personal training schedule (n=970)	209 (49.2%)	245 (45.0%)	1.077 (0.963–1.205)
Used a general training schedule (n=970)	180 (36.5%)	155 (33.0%)	1.070 (0.949–1.206)

**Strength and technique exercises**			
Searched for information on strength exercises (n=961)	246 (59.0%)	374 (68.8%)	0.826 (0.730–0.936)
Searched for information on running techniques (n=961)	218 (51.5%)	274 (50.9%)	1.011 (0.904–1.131)
Performed strength exercises (n=962)	302 (71.9%)	422 (77.9%)	0.865 (0.752–0.995)
Performed running technique exercises (n=984)	255 (58.6%)	283 (51.5%)	1.134 (1.015–1.267)

**Warm-up routine**			
Searched for information on a warm-up routine for runners (n=969)	240 (55.6%)	241 (44.9%)	1.211 (1.080–1.357)
Performed a warm-up routine (n=1 000)	399 (89.7%)	477 (85.9%)	1.155 (0.996–1.340)
Added strength exercises to warm-up routine (n=999)	219 (49.3%)	212 (38.2%)	1.228 (1.092–1.380)

Data expressed as n(%) which indicates the number and percentage of participants within each group that responded “yes” to each action. n represents the total number of participants of which information on the injury-preventive action is available. A runner could undertake one or more preparatory and executional injury-preventive actions.

**Table 3:** Preparatory injury-preventive actions and structural executional injury-preventive actions undertaken by runners over five months of follow-up

	Intervention group	Control group	RR (95% CI)
**Using a (personalised) training schedule (no schedule at baseline)**			
Searched for a training schedule (n=376)	74 (46.3%)	91 (42.1%)	1.074 (0.900–1.283)
Created a personal training schedule (n=792)	134 (38.3%)	142 (32.1%)	1.130 (0.986–1.295)
Used a general training schedule (n=376)	17 (10.6%)	22 (10.2%)	1.021 (0.763–1.365)

**Strength and technique exercises (no exercises at baseline)**			
Searched for information on strength exercises (n=426)	90 (48.4%)	139 (57.9%)	0.845 (0.712–1.003)
Searched for information on running techniques (n=668)	76 (26.4%)	115 (30.3%)	0.923 (0.802–1.062)
Performed strength exercises (n=426)	68 (36.6%)	120 (50.0%)	0.790 (0.669–0.932)
Performed running technique exercises (n=691)	124 (40.8%)	121 (31.3%)	1.208 (1.042–1.400)

**Warm-up routine (no routine at baseline)**			
Searched for information on a warm-up routine for runners (n=194)	45 (53.6%)	37 (33.6%)	1.444 (1.098–1.901)
Performed a regular warm-up routine (n=196)	41 (47.1%)	31 (28.4%)	1.461 (1.084–1.968)
Added strength exercises to warm-up routine (n=195)	28 (32.6%)	19 (17.4%)	1.504 (1.039–2.179)

**Warm-up routine (no extensive routine at baseline)**			
Searched for information on a warm-up routine for runners (n=859)	216 (56.4%)	215 (45.2%)	1.222 (1.083–1.380)
Performed a regular warm-up routine (n=882)	207 (52.8%)	197 (40.2%)	1.257 (1.112–1.421)
Added strength exercises to warm-up routine (n=880)	166 (42.5%)	146 (29.9%)	1.290 (1.127–1.478)

Data expressed as n(%) which indicates the number and percentage of participants within each group that responded “yes” to each action. n represents the total number of participants of which information on the injury-preventive action is available. A runner could undertake one or more preparatory and executional injury-preventive actions.
