# Supplementary material for: The effectiveness of an online intervention in stimulating injury-preventive behaviour in adult novice runners: Results of a randomised controlled trial
Source: S Afr J Sports Med. 2021 Sep 20;33(1):v33i1a11297. doi: 10.17159/2078-516X/2021/v33i1a11297 (PMC9924545; doi:10.17159/2078-516X/2021/v33i1a11297)
Supplement: Supplementary file 1 [file 2078-516X-33-v33i1a11297-s001.pdf]

Appendix A

Development and detailed information content Runfitcheck

Through an expert consultation and research process, two main dimensions in the risk for RRIs were identified: (1) the physical load-taking capacity of runners; and (2) the motivation of runners to achieve their running goals. Across these two domains, runners were classified into four categories: (1) a low physical load-taking capacity and a low goal-orientation; (2) a low physical load-taking capacity and a high goal-orientation; (3) a high physical load-taking capacity and a low goal-orientation; and (4) a high physical load-taking capacity and a high goal-orientation. Depending on their classification, runners directly received tailored advice on the website for achieving optimal running practice. This advice consisted of: 1) advice about the use of a training schedule based on the runner's load-taking capacity and motivation. Runners could also subscribe to receive a personalised running schedule for 6 or 12 weeks (0-3 KM, 0-5 KM, 3-5 KM, 5-10 KM, 10-16 KM). The running schedules were amplified on the runner's load-taking capacity, motivation, and specified time frame until a running event; 2) Four exercises to be performed three times a week to increase strength or improve running technique (plank, skipping, tripling, and leaping sideways on two legs), and the possibility to receive a more varied training programme based on load-taking capacity; 3) An instruction video with voice-over and additional information on performing a warm-up. The warm-up routine consisted of tripling, one-leg skipping, normal skipping, heel-to-buttocks exercise, squats, good mornings, lunges, six different leaping exercises, skating jumps, six different one-leg leaping exercises, squats with arm swing, and high jumps.
